# Effect of oxytocin administration on nerve recovery in the rat sciatic nerve damage model

**DOI:** 10.1186/s13018-015-0301-x

**Published:** 2015-10-14

**Authors:** Bilal Gümüs, Ersin Kuyucu, Oytun Erbas, Cemal Kazimoglu, Fatih Oltulu, Osman Arslan Bora

**Affiliations:** Department of Orthopaedics and Traumatology, Izmir Ataturk Training and Research Hospital, Izmir, Turkey; Department of Physiology, Ege University, Izmir, Turkey; Department of Orthopedics, Katip Celebi University Hospital, Izmir, Turkey; Department of Histology and Embryology, Ege University, Izmir, Turkey; Orthopaedics & Traumatology, Istanbul Medipol University, TEM Avrupa Göztepe çıkışı, No: 1 Bağcılar, Istanbul, Turkey

**Keywords:** Oxytocin, Sciatic nerve, Rat, Nerve regeneration

## Abstract

**Background:**

Growth factors such as nerve growth factor (NGF) and insulin-like growth factor-1 (IGF-1) have been shown to play a role in the healing process of nerve injury. Recent researches have also shown that oxytocin administration activates these growth factors of importance for the healing of nerve tissue. The objective of the present study was to evaluate the effects of oxytocin on peripheral nerve regeneration in rats.

**Methods:**

Twenty-four male Sprague-Dawley rats were underwent transection damage model on the right sciatic nerve and defective damage model on the left sciatic nerve. The animals were assigned to one of two groups: control group or treatment group (received 80 mg/kg oxytocin intraperitoneally for 12 weeks). The sciatic nerve was examined, both functionally (on the basis of climbing platform test) and histologically (on the basis of axon count), 3, 6, 9, and 12 weeks after the injury. Also, stereomicroscopic and electrophysiological evaluations were carried out.

**Results:**

Significantly greater improvements in electrophysiological recordings and improved functional outcome measures were presented in the treatment group at 12-week follow-up. Stereomicroscopic examinations disclosed prominent increases in vascularization on proximal cut edges in the oxytocin group in comparison with the control group. Higher axon counts were also found in this group.

**Conclusion:**

Intraperitoneal oxytocin administration resulted in accelerated functional, histological, and electrophysiological recovery after different sciatic injury models in rats.

## Background

Treatment of peripheral nerve injuries is still challenging and may lead to considerable disability. Besides the treatment of transection injuries, especially those that result in large gaps between nerve ends are particularly troublesome [1–3]. Pharmacologic agents and immune system modulators have been investigated for the purpose of enhancing nerve regeneration [4, 5–10]. However, there is not any drug in clinical use proved to be effective in nerve regeneration. On the other hand, clinical and experimental researches have achieved a remarkable progress in understanding the neurobiology involved in nerve injury over the last decades. A number of molecules such as nerve growth factor (NGF) and insulin-like growth factor-1 (IGF-1) have been shown to enhance nerve regeneration and promote axonal growth rate [8–10].

Oxytocin has been shown to play an important role in wound healing via modulating stress responses. Besides, well-controlled animal studies suggest that oxytocin has a direct influence on the healing process due to its antiinflammatory properties and via modulating some important cytokines [10–13]. Recent researches have shown that exogenous administration of oxytocin increases the plasma levels of NGF and IGF-1. These neurotropic factors have shown to improve nerve healing. Therefore, oxytocin may influence the peripherical nerve healing positively by increasing the plasma levels of these important cytokines [14–17]. Our hypothesis in the present study was that oxytocin would enhance healing following peripheral nerve injury and this may lead to have better outcomes after peripheral nerve injuries especially on orthopaedic and trauma surgery.

In this study, we evaluate the effects of oxytocin administration on peripheral nerve regeneration using an established rat sciatic nerve injury models in male Sprague-Dawley rats.

## Material and methods

Twenty-four male Sprague-Dawley rats weighing approximately 200 g were used in the present study. All animal experiments were performed with the approval of the institutional animal care and use committee. All rats were provided with autoclaved pellets and water ad libitum. The rats were permitted 1 week to acclimate to their environment prior to the study. The animals were housed under a 12-h light/dark cycle. All experimental procedures were underwent under aseptic conditions and were completed in accordance with the National Institutes of Health guidelines on the care and use of laboratory animals for research purposes.

**Fig. 1:**
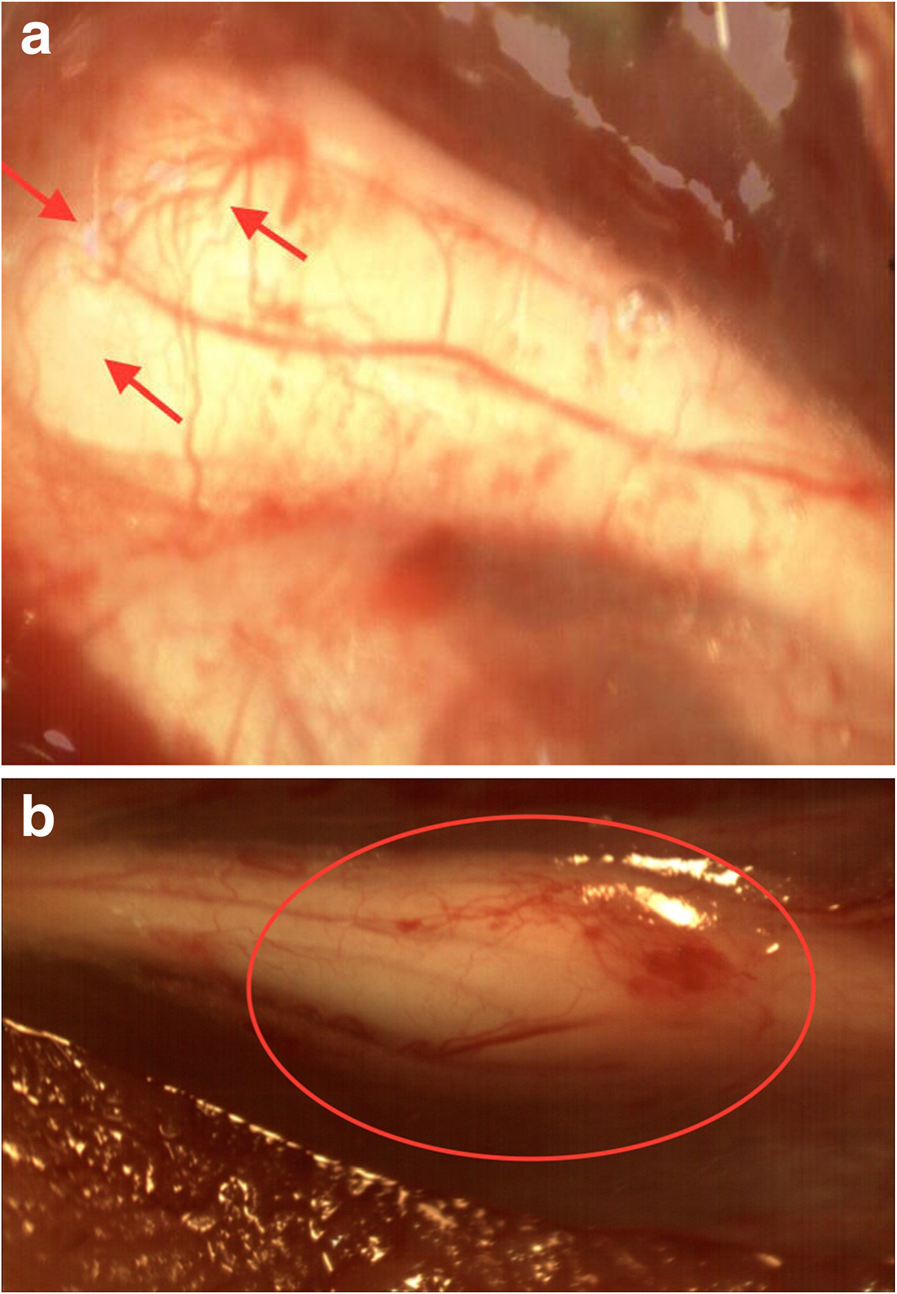
**a** Stereomicroscopic photograph of nerve regeneration in oxytocin treatment group. Axonal sprouting (*arrows*) is pronounced and joins the nerve ends at the third-week follow-up. **b** Completion of nerve healing and maturation of the axonal sprouting at the 12-week follow-up (*circled zone*). The distal stump revealed considerable increase in diameter

The animals were randomly divided into two groups, each group consisting of 12 animals. Group I (treatment group) received 80 mg/kg oxytocin intraperitoneally for 12 weeks (Pituisan 50 ml) [EGE-VET]. Group II (control group) received 1 cm^3^ physiologic serum intraperitoneally for 12 weeks so as to refrain from effects of injection stress. The animals were handled on a daily basis for 1-week period prior to the study (Fig. 1).

### Surgical procedure

All rats were anesthetized with 50 mg/kg Ketamine-HCl (Alfamine®) and 10 mg/kg Ksilazin HCl (Alfazyne) administered intraperitoneally. The surgical site was shaved and was washed with antiseptic solution prior to positioning for surgery. Thereafter, local anesthetic (0.5 mL of 1 % lidocaine hydrochloride) was injected subcutaneously at the surgical site. The sciatic nerve on both sides was approached via semitendinosus-biceps femoris (long head) muscle-splitting incision. After nerve dissection were carried out, transection and defect model was performed at the right and left limbs, respectively. Excision of 10 mm segment from the sciatic nerve was performed in order to obtain a defective nerve model. The wound was closed with 4-0 sutures for the skin. Follow-ups were done at 0, 1, 3, 9, and 12 weeks and one animal in each group were sacrified at each time interval. The nerve regeneration and functional recovery were evaluated by stereomicroscopic observation [SOIF XLB45-B3 + MD30 3.2 MP] and by electrophysiological and histologic analyses.

### Histological evaluation: axon count of sciatic nerve tissue

Following nerve stimulation, the rats were sacrificed. Thereafter, sections measuring 0.5 cm in length were removed from the sciatic nerves, 0.5 cm proximal and 0.5 cm distal to the site of injury. The nerve tissue samples were embedded in paraffin and stained with hematoxylin and eosin blue. Histomorphometric analysis was performed with the use of an SOİF SZM 45 T2 Trinoculer Stero-Microscope with a MD 30 camera for axon counts per unit area. In all animals, the corresponding sections were harvested and processed in a similar manner; the axon counts were performed blinded by a investigator with a light microscope.

### Electrophysiologic evaluation

Investigators who were unaware of the treatment procedure performed the electrophysiological investigations. The results of the EMG (electromyography) tests comprised both amplitude and latency measures. EMG recordings were carried out with the use of MP30 data acquisition and analysis system (Biopac Systems Inc., CA, USA). Proximal electrode was placed slightly distal to the sciatic notch. Distal electrode was placed initially at the second web space of the foot and later on moved distally at the gastrocnemius (GM) muscle to obtain bipolar recording.

### Functional evaluation

The functional assessments of the sciatic nerve recovery were performed by measuring the maximum angle at which the animals were able to climb upon an inclined plane (climbing degrees) which is sensitive in detecting 5-degree difference. The maximum angle at which an animal could support its weight was measured. Climbing level achieved in each group was recorded periodically.

### Statistical analysis

All statistical analyses were conducted using the SPSS 15.0 program. The Student *t* and ANOVA tests were used to determine differences in the parametric values whenever necessary between the groups. The Mann-Whitney *U* test was used to evaluate the nonparametric values. The level of significance was set at *P* < 0.05.

## Results

The mean body weight of the rats before the surgery and at follow-ups was similar in both groups revealing no significant difference.

At one, 3 and 9 weeks follow-ups, higher number of axons were counted in the treatment group among the rats that were sacrified in comparison with the control group. After 12 weeks, all the animals were sacrified and underwent histological assessment. The samples obtained at the right sciatic nerve in oxytocin group presented an ongoing degenerative process. The results at 12 weeks revealed significant difference between the treatment and control group. A significantly higher number of axons were counted distal to the injury in the group of rats that received oxytocin treatment in comparison with the group that received normal saline solution. Table 1 presents the comparison of axon counts of two groups at follow-ups and after completion of the study in detail.

**Table 1:** Body weight measurements (grams)

Follow-up period	Treatment group	Control group	*P* value
	Mean ± SS	Mean ± SS	
Before surgery	195.83 ± 5.15	197.08 ± 4.64	0.187
1 week	203.33 ± 8.88	206.04 ± 10.53	0.342
3 weeks	219.09 ± 25.08	222.73 ± 21.64	0.203
6 weeks	261 ± 36.95	261.5 ± 35.28	0.969
9 weeks	277.78 ± 47.38	277.22 ± 49.09	0.824
12 weeks	284.29 ± 41.98	272 ± 54.01	0.220

Functional assessment of the rats revealed similar results before the operation. Average climbing degree was 70° in each group. Significantly greater climbing degrees were measured at 1 and 12 weeks in the treatment group in comparison of the control group. Table 2 presents the functional assessments in detail at the follow-ups.

**Table 2:** Comparison of groups in terms of axon counting

	Treatment group—right	Treatment group—left	Control group—right	Control group—left
1 week	480.2 ± 27.4	460 ± 13	330.5 ± 19.6	213 ± 24
3 weeks	695.8 ± 41.5	303.33 ± 20.06	327.5 ± 21.7	202.5 ± 18.33
9 weeks	620.3 ± 20	360.30 ± 28	380.4 ± 12.1	320.6 ± 11
12 weeks	443.8 ± 35.9	362.4 ± 16.7	318.6 ± 14.06	249.6 ± 21.4

*P* values were *P < 0.01* and *P < 0.005*, respectively, after comparison of right (transection) and left (defect) models among the groups after 12 weeks

**Fig. 2:**
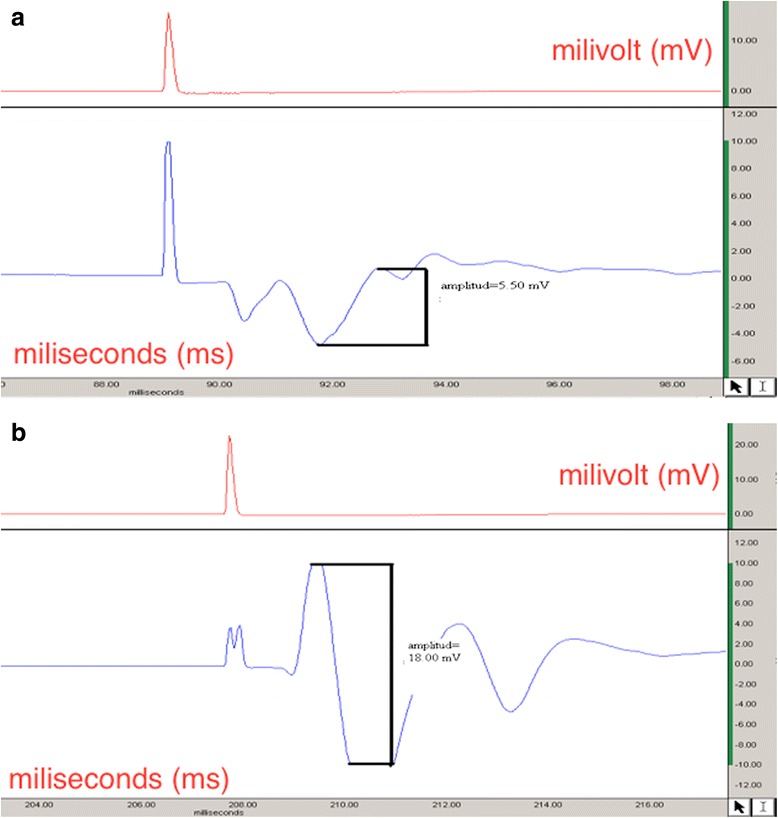
Electromyograph recordings of the right limb (transection model) obtained after 9 weeks. **a** Control group. **b** Treatment group

At postoperative, 1, 3, or 6 weeks group patterns were basically very similar, and there was no EMG response when the distal electrode was located at the second web space. The EMG recordings were carried out via positing the distal electrode at the second web space and GM muscle, respectively, after postoperative 9 weeks. There was no EMG response in both groups when the distal electrode was at the second web space. However, EMG responses were noted at the right side recordings (transection) in the treatment group after replacement of the electrode at the GM muscle at 9-week follow-up. This difference was obtained with regard to amplitude only whereas latency patterns were similar. After 12 weeks, EMG recordings revealed a significant difference in the treatment group with regard to both amplitude and latency in comparison with the control group. The latency recordings were significantly shorter in the treatment group whereas the amplitude measures revealed significantly higher results (Fig. 2).

On the other hand, similar improvements in EMG recordings were seen at the left side (defect model) in the treatment group revealing shorter latency periods and higher amplitude levels when compared with the control group at 12-week follow-up. The results of the electro-diagnostical evaluation are summarized in Tables 3 and 4.

**Table 3:** Functional evaluation via climbing degrees

Follow-up period	Treatment group	Control group	*P* value
	Mean ± SS	Mean ± SS	
1 week	58.33 ± 2.46	55.63 ± 3.99	***0.001 (p< 0.05)***
3 weeks	55.45 ± 2.7	55.91 ± 2.51	0.562
6 weeks	55.5 ± 2.84	56.25 ± 2.75	0.353
9 weeks	57.78 ± 2.64	56.94 ± 3.49	0.258
12 weeks	57.5 ± 5.46	53.57 ± 4.76	***0.002 (p < 0.05)***

**Table 4:** Comparison of EMG recordings between groups

	Treatment group		Control group		*P* values
	Right	Left	Right	Left	R/L
	(Transection)	(Defect)	(Transection)	(Defect)	
9 weeks (amplitude)	12.25 ± 1.57	8.47 ± 1.87	6.99 ± 0.90	6.10 ± 1.53	*0.002*/0.171
9 weeks (latency)	1.25 ± 0.03	1.31 ± 0.04	1.22 ± 0.02	1.27 ± 0.04	0.27/0.269
12 weeks (amplitude)	12.92 ± 2.86	11.17 ± 1.60	8.34 ± 0.42	7.75 ± 0.68	*0.011*/0.039
12 weeks (latency)	1.05 ± 0.01	1.12 ± 0.02	1.16 ± 0.03	1.32 ± 0.03	*0.002*/*0.002*

Stereomicroscopic evaluations of the right sciatic nerve (transection) after the first week revealed that the revascularization at the proximal site of the injury was more prominent. Besides, the diameters of the vessels were larger, and the walls of the vessels were thicker when compared with the control group in the treatment group.

Axonal sprouting was observed in both nerve damage models in both groups after 3 weeks. This sprouting has been observed to reach the distal site of the injury in the transected sciatic nerves in both groups after 9 weeks. Nerve fibers crossed the lesioned region and reached the distal stump joining the nerve stumps in both groups. However, the maturation of the sprouting was more pronounced in the treatment group.

## Discussion

The functional assessment in the present study revealed that oxytocin group presented significant difference with regard to functional outcome at 12-week follow-up. Although we saw a positive effect on nerve regeneration with oxytocin, functional measures failed to demonstrate complete improvement in the recovery from injury. The complete functional recovery might have been observed with prolongation of the experiment time.

Wallerian degeneration is a process of progressive disintegration and demyelination of the distal axonal segment following the damage to the neuron [1]. A pronounced increase in wallerian degeneration was seen in both groups at the first week whereas epinoral fibrosis was significantly higher in control group. An increase in axon count in comparison with the control group was noted at 12 weeks in treatment group. Our findings revealed that nerve transection model was superior to gap model at all follow-ups with regard to histological improvement. The nerve-transection model in oxytocin group presented significant difference in axon count compared with the control group. This difference was also noted in nerve defect model at the 12-week follow-up. This study herein presented that oxytocin enhances a better histological improvement in two different injury models in rats. This improvement was obvious at earlier follow-ups and continued throughout the study.

We assessed that the degree of recovery of sciatic nerve function was assessed via climbing test, which provided a noninvasive and easily quantifiable method in the rat model when both limbs were injured [18]. Goldshmit Y et al. investigated the effect of exercise on axonal regrowth and found positive results with the climbing tests. Our results present the positive effect of oxytocin administration in terms of functional recovery [19]. Our data showed significant difference at 12 weeks in functional evaluation between group comparisons. Moreover, animals able to climb onto the platform from higher grid levels in the treatment group comparable to the preoperative levels. We can speculate that the animals might have shown more improvements if the study duration was prolonged presenting full functional recovery.

Electrophysiologic measurements reflect the functional behavior of the regenerated nerve [1, 2]. Dose F et al. reported the low doses of oxytocin synergism with electrical afferent stimulation after spinal cord injury and similarly [20], our findings indicate that administration of oxytocin significantly provides lower latency recordings and higher amplitude measures reflecting a higher number of normally functioning axons in the treatment group.

Taken together, these data indicate that administration of oxytocin promotes functional recovery and enhances nerve regeneration after sciatic nerve damage in the rat. In the present study, we observed that the improvement in functional recovery was accompanied by significant increases in axon counts in the nerve regeneration site as well as close observations via stereomicroscopic evaluations. At the microscopic level, there was also continuity across the transection site and the defective site as well in the treatment group after 12 weeks. The observed improvement in the axon regeneration is in agreement with the results.

There is increasing evidence that growth factors may act at multiple levels in the regenerative response of nerve healing [8–11]. One such factor affecting multiple cell processes is IGF-1 and NGF. IGF-1 accelerates glucose uptake in the cells, stimulates mitotic activity and cell proliferation, and also inhibits apoptosis. NGF is a member of a family known as neurotrophins that function as signaling molecules. These cytokines are important for the growth, maintenance, and survival of neural cells [11, 12].

It has been well documented in the related literature that oxytocin influences plasma levels of some growth factors that play important role in the healing process of damaged tissues [15, 21]. Luppi et al. have reported that an intravenous injection of oxytocin results in a threefold increase in NGF [15]. Another important study by Gavrilenko et al. revealed that oxytocin resulted in significant differences of wound process characteristics as compared with those ones in control group in the treatment of complex diabetic foot ulcers [19]. The authors presented in their work that oxytocin activates the processes of neovascularization, proliferation of endotheliocytes and histiocytes, resulting in the effective clearance of the wound, and optimal granulation tissue formation. Peterson et al. revealed in his study that oxytocin enhances the survival of musculocutaneous flaps via increasing plasma IGF-1 levels. The authors revealed that this positive effect blocked by administration of an oxytocin antagonist [17]. The present study demonstrated that oxytocin enhances peripheral nerve regeneration in a rat sciatic damage model. This positive impact can be attributed to the benefits of oxytocin in stimulating the nerve growth factors that obviously presented in the literature such as NGF, as well as substantiating other growth factors such as IGF-1, which has positive effects in nerve tissue healing [8, 10].

The current study is not without limitations. The duration of the study could have been prolonged to draw better conclusions with regard to functional evaluation. Also, we did not examine the effect of the oxytocin on the different cytotoxins that promotes nerve regeneration. Future studies should focus on the exact mechanism that provides nerve healing after oxytocin administration. In spite of these limitations, these data provide evidence that oxytocin administration plays a direct role in nerve regeneration

The strengths of this study are the appropriately powered histological measure (stereomicroscope) and analysis of nerve healing in two different injury models. In addition, assessment of nerve regeneration conducted in three different ways consisting of functional, electrophysiologic, and structural evaluations. Understanding these mechanisms may ultimately lead to improvements in peripherical nerve damage.

Our findings show that oxytocin promotes functional recovery and enhances nerve regeneration after transactional and defective peripheral nerve injury in rats compared with controls. The dose response to oxytocin has not been described in the present study Future studies should be performed with different doses to determine clinical application.

In conclusion, these data clearly demonstrated that oxytocin promotes nerve healing in two different rat sciatic damage models. This healing effect can at least in part be ascribed to the fact that oxytocin activates growth factors of importance for the healing of different tissues.
